# Response surface methodology based extraction of *Tribulus terrestris* leads to an upsurge of antilithiatic potential by inhibition of calcium oxalate crystallization processes

**DOI:** 10.1371/journal.pone.0183218

**Published:** 2017-08-28

**Authors:** Jyoti Kaushik, Simran Tandon, Varun Gupta, Jasamrit Nayyar, Surinder Kumar Singla, Chanderdeep Tandon

**Affiliations:** 1 Amity Institute of Biotechnology, Amity University, Noida, India; 2 Amity Institute of Molecular Medicine & Stem Cell Research, Amity University, Noida, India; 3 Department of Biotechnology and Bioinformatics, Jaypee University of Information Technology, Waknaghat, Solan, Himachal Pradesh, India; 4 Department of Chemistry, SD College, Chandigarh, India; 5 Department of Biochemistry, Panjab University, Chandigarh, India; "INSERM", FRANCE

## Abstract

*Tribulus terrestris* has significant antilithiatic efficacy established via both *in vitro* as well as *in vivo* studies and is used in numerous anti-urolithiatic herbal formulations viz. Cystone, Uriflow, Uritone and Neeri. However, to fully utilize its antilithiatic potential, the influence of different extraction parameters on antilithiatic ability of *T*. *terrestris* aqueous extract needs elucidation. Thus, the current study was undertaken using statistically optimized extraction conditions for aqueous extract preparation. Response surface methodology was employed to observe the influence of three variables *i*.*e*. temperature (°C), time (h) and solid: liquid ratio (S: L) on the extraction yield (%) and protein content (mg/g) of *T*. *terrestris* aqueous extract. RSM results revealed that the high S:L ratio, low temperature and reduced incubation time were optimal conditions for aqueous extraction. Under such extraction conditions the protein content reached the value of 26.6±1.22 mg/g and the obtained extraction yield was 27.32±1.62%. The assessment of antilithiatic activity of 4 selected extracts (AE1-4), revealed enhanced nucleation and aggregation inhibition of calcium oxalate crystals with AE1 and AE2, which in addition significantly altered the size and morphology of calcium oxalate monohydrate (COM) crystals compared to AE3 and AE4. *In vitro* cell culture based studies on renal epithelial cells (MDCK, NRK-52E and PK 15) proved that the AE1 showed higher cytoprotective potency by increasing cell viability as compared to the oxalate treated group. The free radical scavenging activity of aqueous extract lowered the reactive oxygen specie’s induced damage and potentially reduced the signals of programmed cell death due to oxalate injury. In addition, modulation of the COM crystal morphology was enhanced by AE1 as compared to AE2. The FTIR and GC-MS analysis of AE1, showed the presence of biomolecules which could aid in the attenuation of lithiatic process. In the light of these results the utility of the RSM approach to fully optimize the antilithiatic potential of *T*. *terrestris* cannot be undermined.

## Introduction

An imbalance between urinary stone promoting and inhibiting factors is predominantly responsible for the formation of renal stones [1], which is a multistep process involving nucleation, crystal growth, aggregation and finally retention of crystals [2]. The current treatment strategies for urolithiasis include shockwave lithotripsy, ureteroscopy and percutaneous stone extractions. However, these treatments are wrought with various side effects. When coupled with the high recurrence rate of stone formation (over 50% in 10 years [3]), it strongly calls for new treatment options.

The lowered side effects associated with herbal medicines has reignited interest in phytomedicine. The Ayurveda system of medicine which is widely followed in India, provides a solid foundation to search new herbal formulations having the ability to act on stones. *Tribulus terrestris* (Zygophyllaceae), locally known as Gokharu or Gokshur is one such plant reported in traditional medicine system *i*.*e* Ayurveda, Siddha, Unani, to have efficacy against urolithiasis. *Tribulus terrestris* is widely used as a diuretic and an antilithiatic agent owing to the various medicinal components such as saponins, flavonoids and alkaloids [4,5,6] as well as the proteins [7]which it possesses. However, to fully realize the antilithiatic potential of *T*. *terrestris* it is necessary to be able to extract these components at their maximum yield. For optimization of extraction conditions, two different approaches viz. “classical” or “statistical” can be employed. The classical approach involves the alteration of one parameter (OFAT) which although is attractive in the scientific sense, as these experiments are easier to perform and less laborious, they suffer from the fact that they are both tedious and time consuming. In addition, this approach cannot effectively discriminate between the cumulative effect amongst the selected parameters. As with any extraction procedure there are a large number of variables which can impact the extraction parameters as well as the yield and therefore, there is a requirement of a specific system which can take into account every single variable on the overall efficiency of the procedure. Response surface methodology (RSM) is a statistical approach which has been widely established to improve the extraction process with a minimal input of experimental data [8]. With the help of RSM the influence of various conditions on the extraction procedure can be simulated, both individually and through their cumulative interactions, thereby giving dry lab values for optimization of wet lab procedures [9,10].

To optimize the extraction condition for *T*. *terrestris* we chose certain variables which could impact the extraction conditions. The choice of the variables was based upon previous studies carried out in our lab using various medicinal plants, wherein we had seen that temperature, time and the solid liquid ratio all affected the extraction process. We selected the extraction solvent as water since the medicinal plants used in the ayurvedic/traditional system of medicine are usually ground into a paste using aqueous solvent, usually water, and more importantly these can be readily used as oral formulations whereas others need to have the solvent removed and then resolubilized before consumption. The rationale for the current study was therefore to statistically optimize different process parameters viz. temperature, solid to liquid ratio (S: L) and time, by employing RSM, in order to improve the yield and protein content of *T*. *terrestris* aqueous extract. We have earlier reported that it is not only the secondary metabolites such as saponins, flavonoids, terpenoids etc, but also proteins present in the plants which contribute to the antilithiatic activity [11]. The optimized aqueous extract, which we have referred to as AE (1 through 4), was evaluated for its antilithiatic potential by assessing inhibition of calcium oxalate crystallization. Urolithiasis is a multistep procedure wherein the initial process of crystal formation involves (i) nucleation followed by ii) aggregation of calcium oxalate crystals. These crystals can then lead to iii) cytotoxicity by damaging the renal epithelial cell membrane leading to iv) inflammation which further perpetuates the damage. It is known that exposure to high levels of oxalate crystals results in injury and evokes a series of intracellular reactions and events in renal epithelial cells including the production of reactive oxygen species. Oxalate injury also leads to the expression of several proteins such as bikunin, osteopontin, prostaglandins and monocyte chemoattractant protein 1 (MCP-1) which contribute to the inflammation process [12]. Retention of calcium oxalate monohydrate (COM) crystals on cellular surface is a key event for the development of kidney stones owing to the interaction between cell membranes and oxalate crystals, mediated by surface of crystal and surface of renal cell membrane [13]. To quantitatively assess whether our RSM optimized extracts were playing a role in the inhibition of the crystal formation thereby leading to diminution of damage, we carried out a series of assays for (i) nucleation and (ii) aggregation. In addition as cells damaged by COM would lead to (iii) loss in cell viability and the ability of the extracts to (iv) scavenge the free radicals generated were also evaluated.

The findings of our current study systematically proves the antilithiatic efficacy of the aqueous extracts of *T*.*terrestris* obtained through the RSM approach, based on inhibition of oxalate crystal damage to renal epithelial cells. To the best of our knowledge and literature search, no previous reports are available on application of RSM for T. terrestris to explore its antilithiatic potential.

## Material and methods

### Plant sample

The dried and matured fruits of *Tribulus terrestris* appear as woody burrs consisting of 5 wedge shaped segments and each segment having 2 unequal pair of spines. The sample of *Tribulus terrestris* which was used in this study was obtained from “Natural Remedies Pvt. Ltd.” at Bangalore, India. The voucher specimen (No: AGR 820) of the same is available with the company.

### Optimization of extraction procedure

To optimize the extraction condition, we needed to select certain variables which could have a direct bearing on the extraction conditions. The choice of the variables made were based upon previous studies carried out in our lab using various medicinal plants, wherein we had seen that temperature, time and the solid liquid ratio are key parameters that impact the extraction process. In this study, the three process parameters were selected for the extraction of *T*.*terrestris* namely, temperature (°C), time (h) and S: L ratio (g/mL) and were statistical optimized by employing RSM.

To examine the effect of selected parameters, a 2^3^ full factorial central composite design (CCD) was employed in RSM to evaluate the effect of the interactions between the process variables and proposed a total of 20 experiments with 8 trials for factorial design, 6 trials for axial point and 6 replicate trials for central point through Design Expert software version 8.0.7.1 (Stat-Ease, Inc., USA). The details of designed experiments are provided in Table A in S1 Appendix (supporting information). Further, the aqueous extraction of *T*. *terrestris* was performed in triplicates for all the designed experiments. The extracts obtained were filtered through muslin cloth as well as Whatman filter paper and the filtrates were lyophilized to obtain the dried powder. Each lyophilized sample was weighed and subjected to protein estimation [14]. The average values of each experiment were recorded for two different responses viz. extraction yield (%) and protein content (mg/g) to investigate the cumulative effect of selected variables. The aqueous extracts showing desirable yield and protein content, were selected. A total of 4 extracts were then subjected to physico-chemical analysis and in vitro assays and designated

AE1 (temp.23.50°C, time 19.50 h and S:L 1g/12 mL)
AE2 (temp.4.16°C, time 19.50 h and S:L 1g/12 mL)
AE3 (temp.35°C, time 36 h and S:L 1g/20 mL)
AE4 (temp.35°C, time 3 h and S:L 1g/20 mL)

### Evaluation of antilithiatic activity

The ability of the RSM optimized extracts to inhibit the multistep process of crystal formation was evaluated using *in vitro* assays addressing the formation and growth of the crystals as well as its ability to scavenge free radicals. The antilithiatic ability was further validated in renal epithelial cells from 3 mammalian species to ascertain whether a common mechanism existed.

### Inhibition assays for calcium oxalate monohydrate crystals formation

The various concentrations of each extract AE1, AE2, AE3, or AE4, were tested for their inhibitory potency on the processes of nucleation and aggregation, using standardized procedures previously published [11]. The ayurvedic drug Cystone (Himalaya Herbal Healthcare) (1 mg/mL) was taken as the positive control. The percentage inhibition produced by *T*. *terrestris* extract was calculated as by the formula [1-(Tsi/Tsc)] X 100, where Tsc was the turbidity slope of the control and Tsi the turbidity slope in the presence of the inhibitor (test sample).

### Radical scavenging activity

The ability of the extracts to scavenge free radicals and hence contribute to the inhibition of the inflammatory process was evaluated by the free radical scavenging 1,1-diphenyl-2-picrylhydrazyl (DPPH) radical-scavenging activity [15].

### Modulation of crystal morphology

Based on the ability of the extracts to inhibit the nucleation and aggregation steps of crystal formation as well their radical scavenging activity, AE1 and AE2 were selected for further evaluation of antilithiatic potential. The effect of individual extract at a concentration of 1mg/mL, on calcium oxalate (CaOx) crystal morphology was evaluated [16,17]. Stock solutions of CaCl_2_ (12.75 mM) and Na_2_C_2_O_4_ (2.25 mM) were used for the preparation of calcium oxalate crystals. 50 μl CaCl_2_ (12.75 mM), 50μL Na_2_C_2_O_4_ (2.25 mM) and 50 μL distilled water were added in each well of a 96 well plate to obtain final concentration of CaCl_2_ (4.25 mM) and Na_2_C_2_O_4_ (0.75 mM) and taken as control. 50 μL of test sample, 50 μL CaCl_2_ (12.75 mM) and 50 μL Na_2_C_2_O_4_ (2.25 mM) were mixed gently and incubated at 37°C for 45 min. The crystal morphology was observed at 40X magnification under an upright polarized microscope (Olympus CH2i). Samples were coated with electrons and scanning electron microscopic images were captured by ZISS (EVO18). Images were captured for each concentration of test material as well as for blank and multiple fields were assessed.

### Assessment of lithiatic potential in renal epithelial cells

In order to evaluate the antilithiatic ability of the selected extracts on renal cells, experiments were conducted using renal tubular epithelial cell lines from 3 different mammalian species, dog, rat and pig i.e Madin-Darby Canine Kidney (MDCK), Normal Rat Epithelial Kidney (NRK-52E) and Porcine Kidney (PK 15), respectively. This was done to ascertain whether a common underlying mechanism for attenuating the damage was prevalent across the various species. MDCK and NRK-52E cell lines were obtained from NCCS (National Centre for Cell Science), India and PK 15 from NRCE (National Research Centre on Equines), Hisar, India. Cell lines were maintained in Dulbecco’s Modified Eagle’s Medium (DMEM) supplemented with 10% Fetal Bovine Serum (FBS) and 1% Penicillin (100 units/mL)-Streptomycin (10,000μg/mL). Cells were cultured in tissue-culture treated flasks at 37°C under 5% CO_2_ in humidified chambers.

### Sample preparation for cell line studies

For the preparation of the master stock solution the lyophilized powder of aqueous extract of *T*. *terrestris* (1 mg/mL), was dissolved in dimethyl sulfoxide (DMSO) [final concentration of DMSO did not exceed 0.4% (v/v)]. Stock solution was filtered through 0.22 μm syringe filter. Subsequently serial dilutions of (10–100 μg/mL) of the extracts, AE1 and AE2, were prepared in DMEM serum free media. Cystone (50 μg/mL) was taken as the positive control.

### Oxalate crystal preparation and exposure to the renal epithelial cell lines

Sodium oxalate (10 mM) stock solution was prepared and diluted to 2 mM in serum-free medium. MDCK, NRK-52E and PK 15 cell lines were exposed to 2 mM sodium oxalate for 48 h along with the various concentration of either AE1 or AE2 of *T*.*terrestris* or Cystone (positive control).

### Cell viability assay

For cell viability assay, cells were seeded at a density of 1 ×10^4^ cells/well in 96 well microplate and incubated at 37°C and 5% CO_2_ in humidified chamber till confluence reached 75%. The cells were then treated with 2 mM sodium oxalate, in the absence and presence of AE1 and AE2, at concentrations of 10, 25, 50 and 100 μg/mL. After 48 h treatment, 20 μL of MTT reagent (concentration 0.5 mg/mL) was added to each well and further incubated at 37°C for 4 h. Metabolically active cells could reduce MTT with the help of enzyme Succinate dehydrogenase and form purple colored insoluble formazan. To dissolve these formazan crystals after the 4 h incubation, supernatant was replaced with 200 μL of DMSO (100%) and microplate was incubated at room temperature for 15–20 min. After incubation, gentle mixing was done and absorbance values were determined at 570 nm test wavelength and a 630 nm reference wavelength to evaluate the cell viability using a microplate reader (Model 680, Bio-Rad) [18].

### ROS, hydrogen peroxide (H_2_O_2_) assay

Cellular H_2_O_2_ level was measured by using commercially available PerXOquantTM Quantitative peroxide assay kit (Thermo Scientific). For estimation of cellular H_2_O_2_ level, cells were cultured in 96 well microplate by seeding the cells at a density of 1×10^4^ cells/well at 37°C and 5% CO_2_. The cells were injured with oxalate (2 mM) and cotreated with test concentrations of AE1 and AE2 for 48 h. After this treatment period, 50 μL of supernatant from each well was transferred to a fresh flat bottom 96-well microplate and 200 μL of working reagent (WR: prepared by diluting from the stock solution of Reagent A and Reagent B, supplied with kit and as per instructions provided) was added to the wells containing samples, standard and reagent blank and incubated for 15 min at room temperature. Absorbance was measured spectrophotometrically at 595 nm by using microplate reader (Model 680, Bio-Rad).

Cellular H_2_O_2_ concentration was calculated by using a standard curve (prepared by taking various concentrations of H_2_O_2_ ranging from 0 to 100 μM.). Cellular H_2_O_2_ levels were expressed in percentage and calculated by following formula.

$$\text{Percentage }\left(\%\right){\text{ H}}_{2}{\text{O}}_{2}=\left[\text{Absorbance of Sample}/\text{Absorbance of Control}\right]\;\times \;100$$

### Crystal adhesion and morphology modulation on renal cells

Crystal adherence and morphology modulation potencies of AE1 and AE2 were evaluated by polarization microscopy and scanning electron microscopy (SEM) analysis. Cells were seeded at a density of 2 ×10^5^ cells/well, on sterilized coverslip in a 6 well tissue culture plate and incubated at 37°C and 5% CO_2_. The cells were then exposed to 2 mM sodium oxalate and treated with AE1 and AE2 by replacing media of each well with various concentrations (10 μg/mL-50 μg/mL) and incubating at 37°C and 5% CO_2_ in humidified chamber. After treatment period, medium was removed and washing of cells was carried out with 1X phosphate buffer saline (PBS) to remove non-adherent crystals from the cell surface. The cells were subsequently fixed by using freshly prepared 4% paraformaldehyde for 30 min and then washed twice with 1X PBS. The cell surface adhered crystals were inspected at a magnification of 20 X using a polarization microscope (BX53, Olympus Corporation, Japan).

### Microscopic analysis of cell death

Acridine orange (AO) and ethidium bromide (EB) double staining assay was performed to observe the characteristic changes of programed cell death (apoptosis and necrosis). Cells were seeded at a density of 2 ×10^5^ cells/well in a 6 well tissue culture plate and incubated at 37°C and 5% CO_2_ in humidified chamber till confluence reached 75% and exposed to 2mM sodium oxalate and treated with test concentrations (10 μg/mL-50 μg/mL) of AE1 and AE2 by replacing media of each well and incubated at 37°C and 5% CO_2_. After treatment period, cells from each well were trypsinized and finally resuspended in 50 μL of cold 1X PBS. For each test sample, 10 μL of cell pellets were stained with 10 μL of fluorescent dye mixture containing equal volume of AO (100 μg/mL) and EB (100 μg/mL) for 2 min at room temperature in the dark. Freshly prepared stained cell suspension (10 μL) was placed on a glass slide, covered with a coverslip and then photographed within 20 min, under fluorescence microscope (Nikon eclipse, Ti) at a magnification of 20 X [19].

### Phytochemical screening and characterization of AE1

For the qualitative and quantitative evaluation of the various secondary metabolites present in the extracts (AE1-4) obtained through the RSM procedure, standardized methods were followed [20]. The details are given in the supporting information in S2 Appendix. As AE1 exhibited potent antilithiatic ability, it was subjected to further characterization using FTIR and GC-MS analysis to ascertain the presence of bioactive components present.

### Statistical analysis

Data were expressed as mean ± SD values of three independent experiments (each in triplicate) and analyzed by ANOVA (p<0.05) to estimate the differences between values of extracts tested using the software GraphPad Prism version 6.01

## Results

### Optimization of extraction of *T*. *terrestris* aqueous extracts using RSM

In this study, our first objective was to statistically optimize the extraction procedure of *T*. *terrestris* through RSM approach. Basically, RSM is a collective package of mathematical and statistical techniques which owing to the ease of use and accuracy, is an important application for the development of new products, process optimization and to improve the quality of existing products. Therefore, the three selected process parameters for the extraction of *T*.*terrestris*, temperature (°C), time (h) and S: L ratio (g/mL) were statistical optimized by employing RSM.

Multiple regression analysis on the data obtained was performed by using design expert (STATEASE Inc., USA). The responses followed a quadratic model that can be represented by the following equation

$$\text{Extraction yield }\left(\%\right)=+12.34-1.79\text{A}-2.76\text{B}+4.15\text{C}-0.79\text{AB}+3.86\text{AC}+0.42\text{BC}+3{.64\text{A}}^{2}+2{.20\text{B}}^{2}-0{.033\text{C}}^{2}$$

$$\text{Protein content }\left(\text{mg}/\text{g}\right)=+22.86-2.84\text{A}-1.28\text{B}+4.52\text{C}-0.26\text{AB}+2.89\text{AC}+1.59\text{BC}+0{.53\text{A}}^{2}-1{.75\text{B}}^{2}-3.69\text{C}$$

Where ‘A’ is temperature (°C), ‘B’ is time (h) and ‘C’ is S: L ratio (g/mL).

The analysis of variance (ANOVA) by F-test (Fisher’s test) was used to calculate the statistical significance of regression, for the significance of model equation. The detailed analysis is shown in Figures A and B in S1 Appendix along with the interaction amongst the variables impacting the yield and protein content illustrated through 3D plots in Figures C and D in S1 Appendix.

Analysis of RSM optimization experiments revealed that lowered temperature, high S:L ratio and decreased incubation time promoted best recovery both in terms of percent extraction yield and protein content in aqueous extract of *T*. *terrestris*. The bioactivity of any extract can be attributed to its constituents which could be present in varying quantities depending on the extraction conditions. The 4 extracts chosen for further analysis reflected those with either the highest extraction yield (AE4), or protein content (AE2) or conditions with low yield but reasonable protein content (AE1) and finally those with intermediate yield and protein content (AE3). The range of selected parameters along with mean values of extraction yield and protein content observed through experiments are shown in Table 1.

**Table 1:** Process variables and their experimental response in the selected extracts.

Aqueous extract	Extraction Variables			Yield (%)	Protein Content (mg/g)
	Time(h)	Temperature(°C)	S:L (g/mL)		
AE1	19.5	23.50	1:12	12.43±0.93	23± 1.77
AE2	19.5	4.16	1:12	25.2± 1.05	26.6±1.22
AE3	36	35	1:20	21.43± 0.98	22.6±2.13
AE4	3	35	1:20	27.3± 1.62	23.6 ± 0.87

The accuracy of the analysis of the generated model through the RSM approach was evident on comparing the experimental values of protein content and extraction yield with the predicted values. The range of selected parameters along with mean values of extraction yield and protein content observed through experiments and those predicted by RSM are given in Table A in S1 Appendix.

### Assessment of antilithiatic potential

The main objective of our study was to evaluate the ability of optimized extracts to attenuate the various steps of the complex process of stone formation involving nucleation and aggregation of calcium oxalate crystals, subsequently leading to cell-crystal adhesion and cellular injury and inflammation, resulting in perpetuation of damage. In order to assess these cascade of events, we systematically studied the ability of these optimized extracts on the extent of nucleation and aggregation of CaOx crystals, along with their ability to modulate the morphology of CaOx crystals using polarization and scanning electron microscopy and validating these findings in oxalate injured renal epithelial cells.

### Effect of RSM optimized aqueous extracts on CaOx crystallization

**Fig 1:**
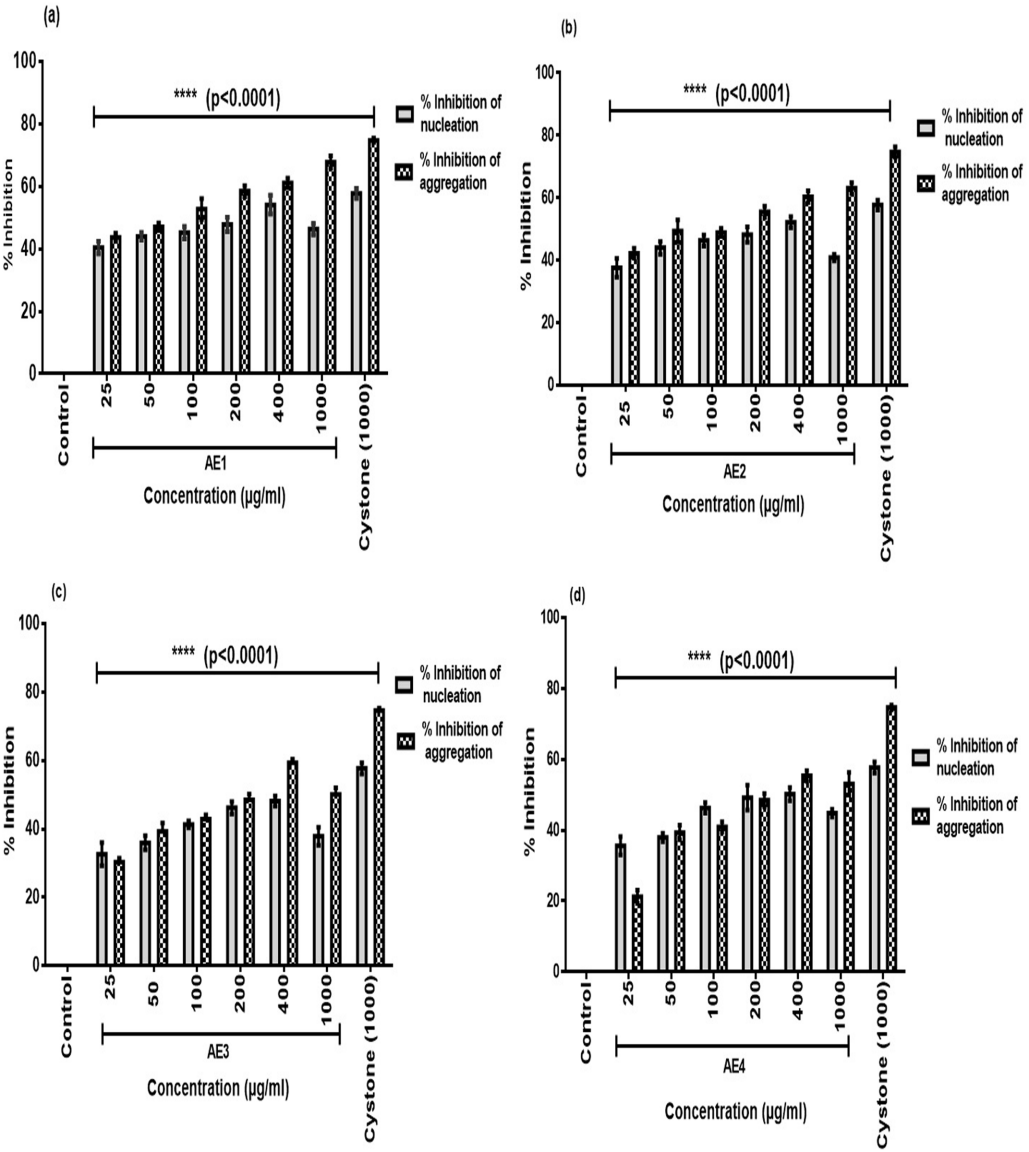
Inhibition of calcium oxalate crystal nucleation and aggregation potency by aqueous extracts. Values are means ± SD ****<0.0001 indicate the significant difference in percentage inhibition of nucleation and aggregation w.r.t control. Cystone (1000 μg/mL) considered as positive control.

Using the RSM approach, we selected 4 extracts which were screened for their ability to inhibit the process of CaOx crystallization. As nucleation and aggregation are initial events leading to the process of crystal formation, *in vitro* crystallization assays were performed for the evaluation of percentage inhibition of nucleation and aggregation by AE1, AE2, AE3 and AE4. There was a steady and significant dose dependent increase in percentage inhibition of nucleation by AE1 from 25 to 400 μg/mL with the maximum inhibition of 54.1% (p<0.0001) followed by a dip at 1000 μg/mL (Fig 1a) with respect to controls (untreated). However, the percentage inhibition of aggregation increased continuously in a dose dependent manner with a maximum inhibition of 67.6% (p<0.0001). Similar trends of percentage inhibition of nucleation and aggregation were observed for AE2 (b), AE3 (c) and AE4 (d).

### DPPH radical-scavenging activity

**Fig 2:**
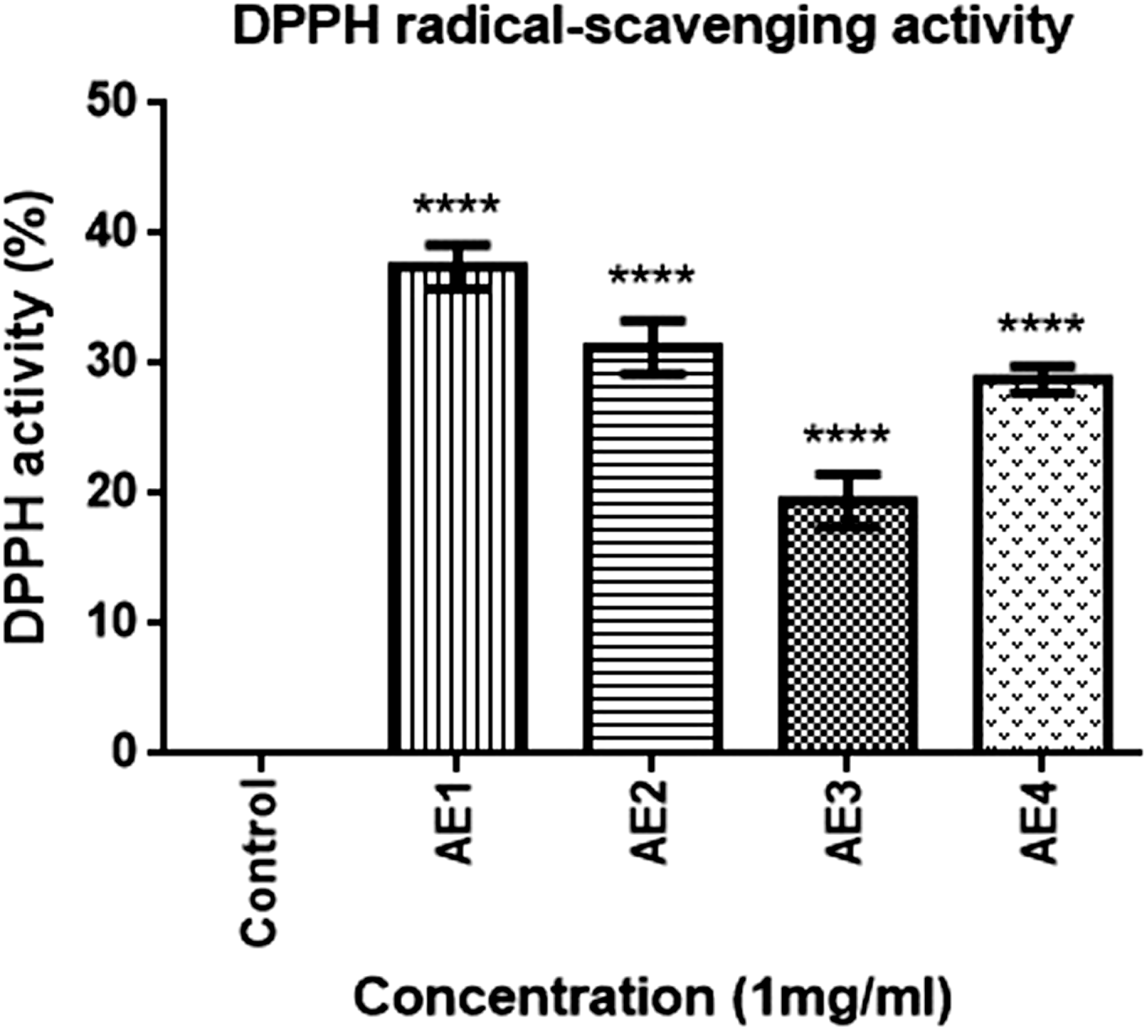
DPPH free radical scavenging activity of optimized aqueous extracts of AE1, AE2, AE3 and AE4. **** p<0.0001 indicates the significant difference in free radical scavenging activity with respect to control.

As in the ongoing process of crystal-cell interaction has been shown to cause a number of perturbations which include the formation of reactive oxygen species, which can then lead to mitochondrial damage and inflammation it was necessary to evaluate the radical scavenging ability of the optimized extracts. The ability to quench DPPH free radicals was noted in all aqueous extracts of *T*.*terrestris* as indicated by the increase in the percentage inhibition with respect to the control. However, the scavenging activity of aqueous extracts of *T*.*terrestris* varied with different extraction parameters as was evident from the results shown in Fig 2. The extraction conditions significantly (p<0.0001) impacted the free radical scavenging activity of extracts, wherein AE1 showed highest antioxidant potential (37.5%) followed by AE2 (31.2%), AE3 (19.5%) and AE4 (28.8%), with respect to control. The results obtained for the extracts were comparable to the composite positive control, Cystone.

### Validation of CaOx morphology attenuation efficiency by optimized aqueous extracts

**Fig 3:**
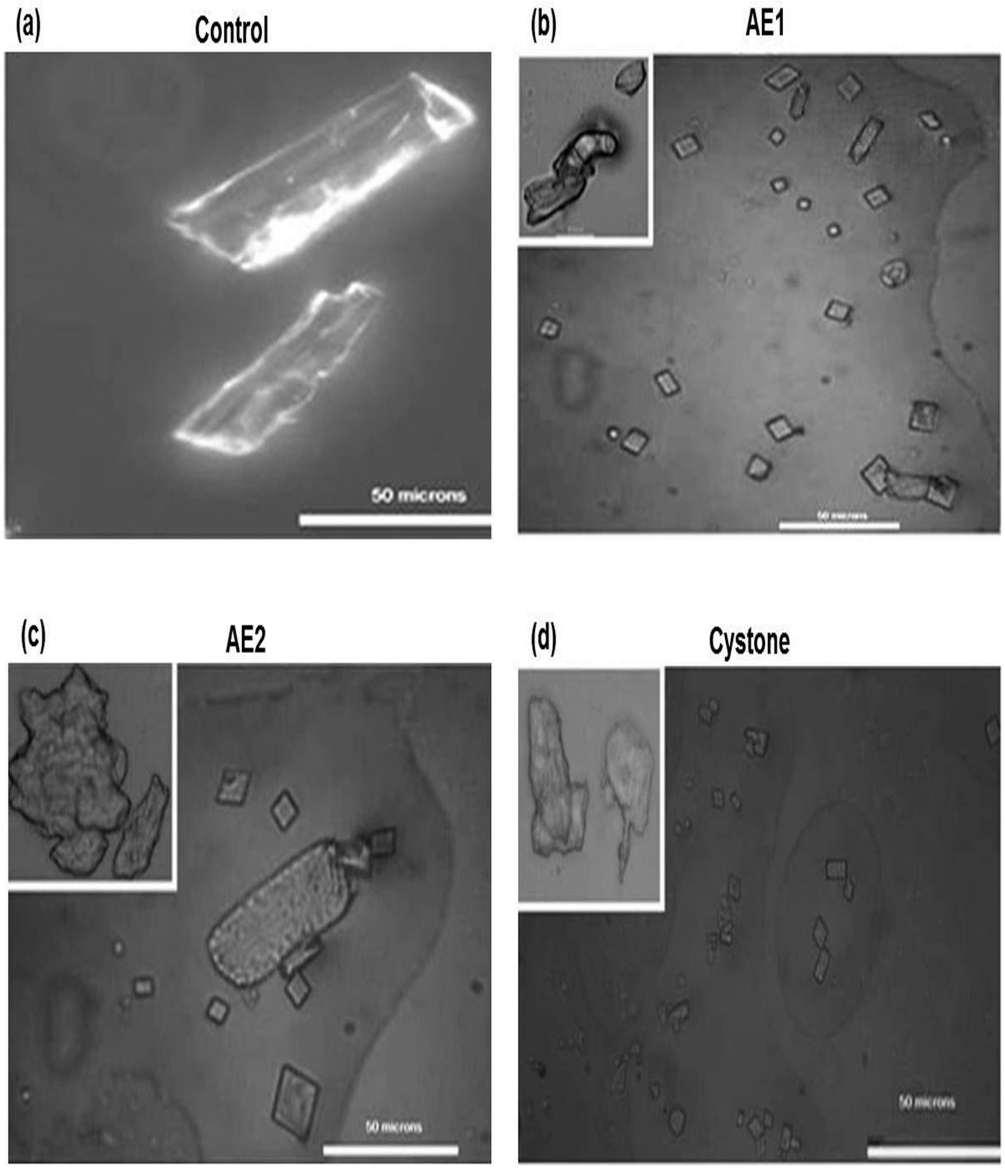
Polarization photomicrographs depicting effect of *T*.*terrestris* on morphology of calcium oxalate crystals. Polarization microscopic images show the morphological changes in calcium oxalate crystals by AE1 (1 mg/mL) and AE2 (1 mg/mL) (Fig. 3b- Fig. 3c) treated groups with respect to untreated group *i*.*e* control (Fig.3a). Cystone (1 mg/mL) treated group (Fig.3d). Images were captured at 40 X magnification; scale bar 50 μm. Multiple fields were assessed.

AE1 and AE2 were found to be most effective based on the preliminary results of attenuation of the nucleation and aggregation process of crystallization as well as the ability to scavenge the free radicals. Modulation and transition of COM crystals to their less damaging COD form can have a significant effect on the cell crystal interaction phenomenon. Therefore, we evaluated this ability which could contribute to the antilithiatic potential of the extracts by studying modulation of the thermodynamically metastable form of calcium oxalate (CaOx) crystals i.e COM crystal morphology. Polarization micrographs Fig 3a showed hexagonal, elongated COM crystals having shining reflective surfaces and sharp pointed edges present in the control group (no treatment). There was a marked conversion from the hexagonal COM crystals to the thermodynamically unstable and more soluble form i.e. CaOx dihydrate (COD) crystals, upon treatment with AE1 (Fig 3b) and this modulation of morphology was superior as compared to crystals exposed to AE2 (Fig 3c). Although some COM crystals were observed they were fewer in number, smaller sized, with a distorted crystal surface and hence less bright.

**Fig 4:**
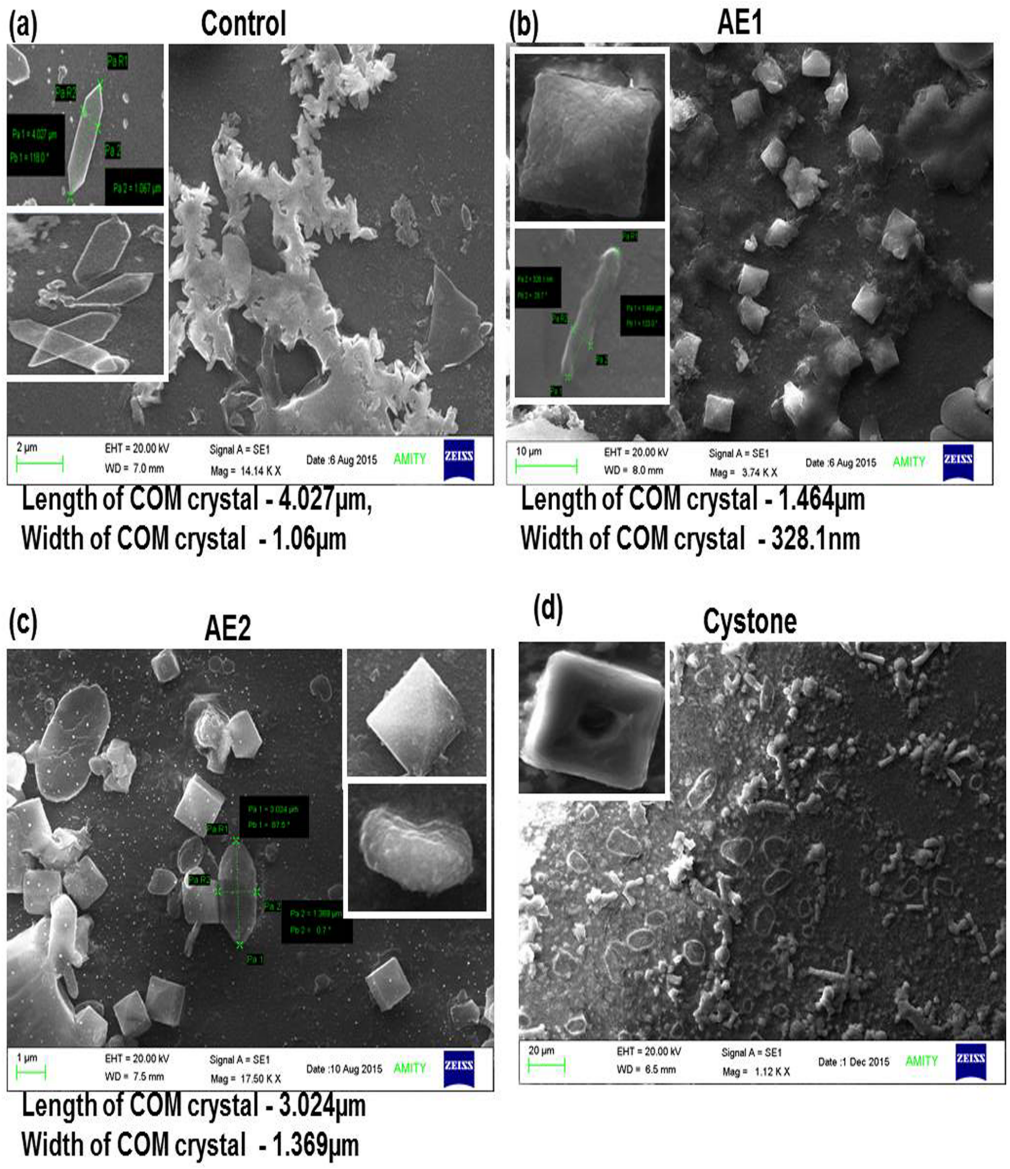
SEM assessment of *T*.*terrestris* modulation of calcium oxalate crystal morphology. Scanning electron photomicrographs show significant modulation in morphology and reduction in size of calcium oxalate crystals formed in AE1 (1 mg/mL) and AE2 (1 mg/mL) (Fig.4b -c) with respect to untreated group *i*.*e* control (Fig.4a). Cystone (1 mg/mL) treated group (Fig.4d). Multiple fields were assessed by scanning electron microscope at following magnifications:
aControl—(4.25 mM Ca^2+^ and 0.75mM Ox) at magnification 14.14 K.X, scale bar 2 μm.b(4.25 mM Ca^2+^ and 0.75 mM Ox) treated with AE1 (1 mg/mL); magnification 3.74 K.X, scale bar 10 μm.c(4.25 mM Ca^2+^ and 0.75 mM Ox) treated with AE2 (1 mg/mL); magnification 17.50 K.X, scale bar 1 μm.d(4.25 mM Ca^2+^ and 0.75 mM Ox) treated with Cystone (1 mg/mL) was taken as positive control at magnification 1.12 K.X, scale bar 20 μm

To verify the results of polarization microscopy and for evaluating the effect of AE1 and AE2 on their ability to reduce the size of COM crystals with respect to the control, scanning electron microscopy was carried out. Aggregation of hexagonal COM shaped crystals with pointed and sharp edges in control was noted (Fig 4a), with the insert image indicating the length (4.03 μm) and width (1.06 μm) of COM crystals. Magnified SEM micrographs (Fig 4b) indicated marked alterations in the morphology of COM to COD shaped crystals, in the presence of AE1 along with remarkable reduction in length (1.46 μm) and width (328.1 nm) with respect to the control. Although AE2 also altered the morphology of COM crystal to COD crystals (Fig 4c) along with reduction in length (3.02 μm) however, changes in width (1.37 μm) were negligible with respect to control.

### Cytoprotective effects of optimized extracts

**Fig 5:**
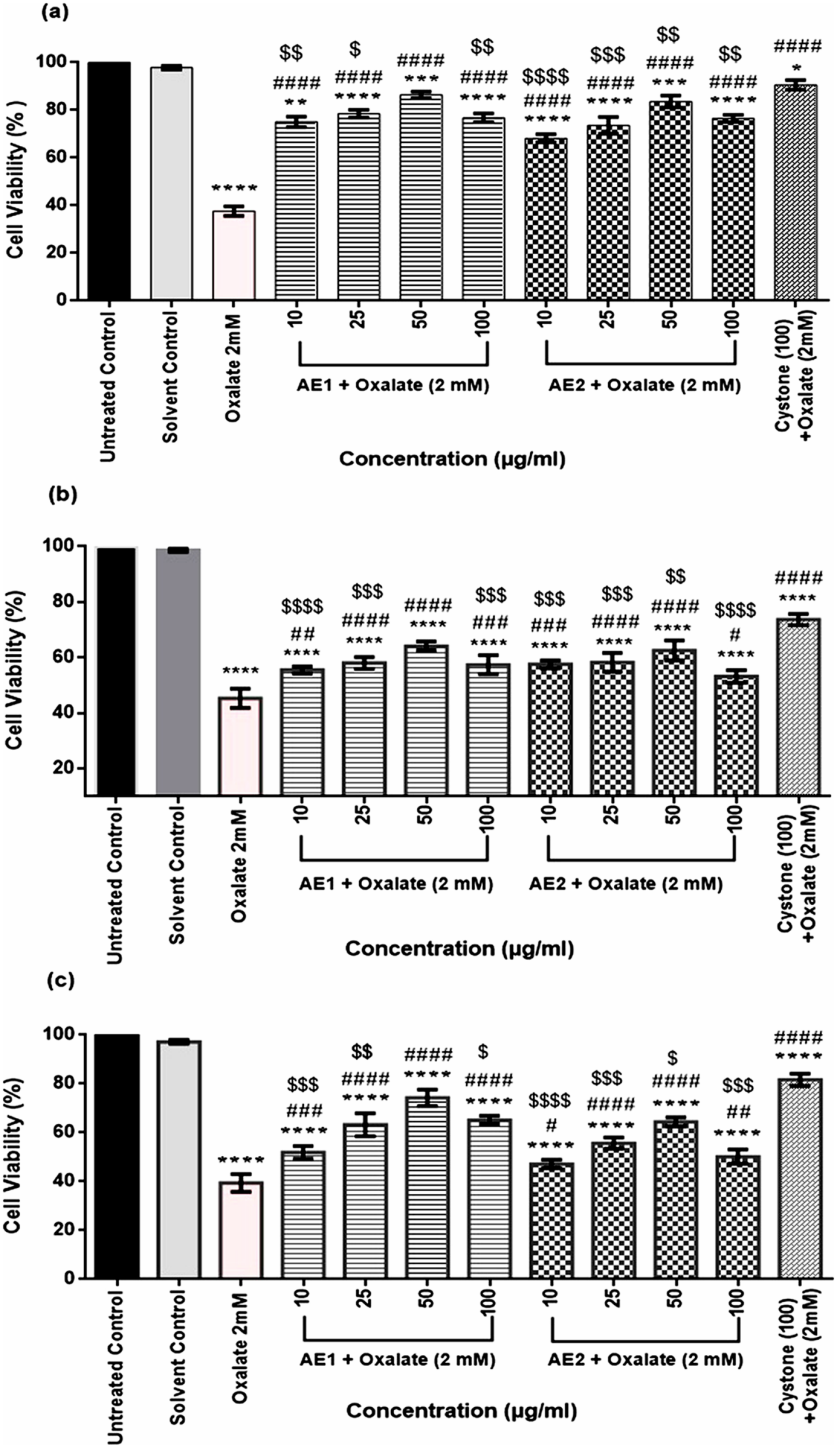
Cytoprotective effect of aqueous extracts of *T*.*terrestris* against oxalate injured renal epithelial cell lines (MDCK, NRK-52E and PK 15). Cytoprotective efficacy of AE1 and AE2 against oxalate injured a: MDCK cells. b: NRK-52E cells. c: PK 15 cells. Cells treated with serum free media were considered as untreated control and cystone at concentration 100 μg/mL was taken as positive control.*p<0.05,**<0.01,***<0.001,****<0.0001indicates significant difference in percentage of cell viability w.r.t to untreated control. ^#^p<0.05, ^##^<0.01, ^###^<0.001, ^####^<0.0001 indicates significant difference in percentage of cell viability w.r.t to oxalate (2 mM) injured group. ^$^p<0.05, ^$ $^<0.01, ^$ $ $^<0.001, ^$ $ $ $^<0.0001 indicates significant difference in percentage of cell viability w.r.t to Cystone treated group *i*.*e* positive control.

To examine the cytoprotective efficacy of aqueous extracts AE1 and AE2, against oxalate injury, the viability of the renal epithelial cell lines (MDCK, NRK-52E and PK 15) following treatment for a period of 48 h, was assessed by the MTT assay. As seen in Fig 5, exposure to oxalate significantly reduced the cell viability from 100% in untreated cells to 37.7% in MDCK (Fig 5a), 44.2% in NRK-52E (Fig 5b) and 37.9% in PK 15 (Fig 5c), respectively. Interestingly cotreatment with test concentrations of AE1 and AE2 resulted in an increase in cell viability w.r.t oxalate treated group with maximum viability seen on treatment with 50 μg/mL of the extracts, in all the selected cell lines and was comparable with the positive control, Cystone (100 μg/mL). As the greatest cytoprotective efficacy of aqueous extracts AE1 and AE2 of *T*.*terrestris* was observed at 50 μg/mL, it was chosen as the optimum concentration for in vitro studies.

### Reduced oxidative stress by optimized aqueous extracts in oxalate injured renal epithelial cells

**Fig 6:**
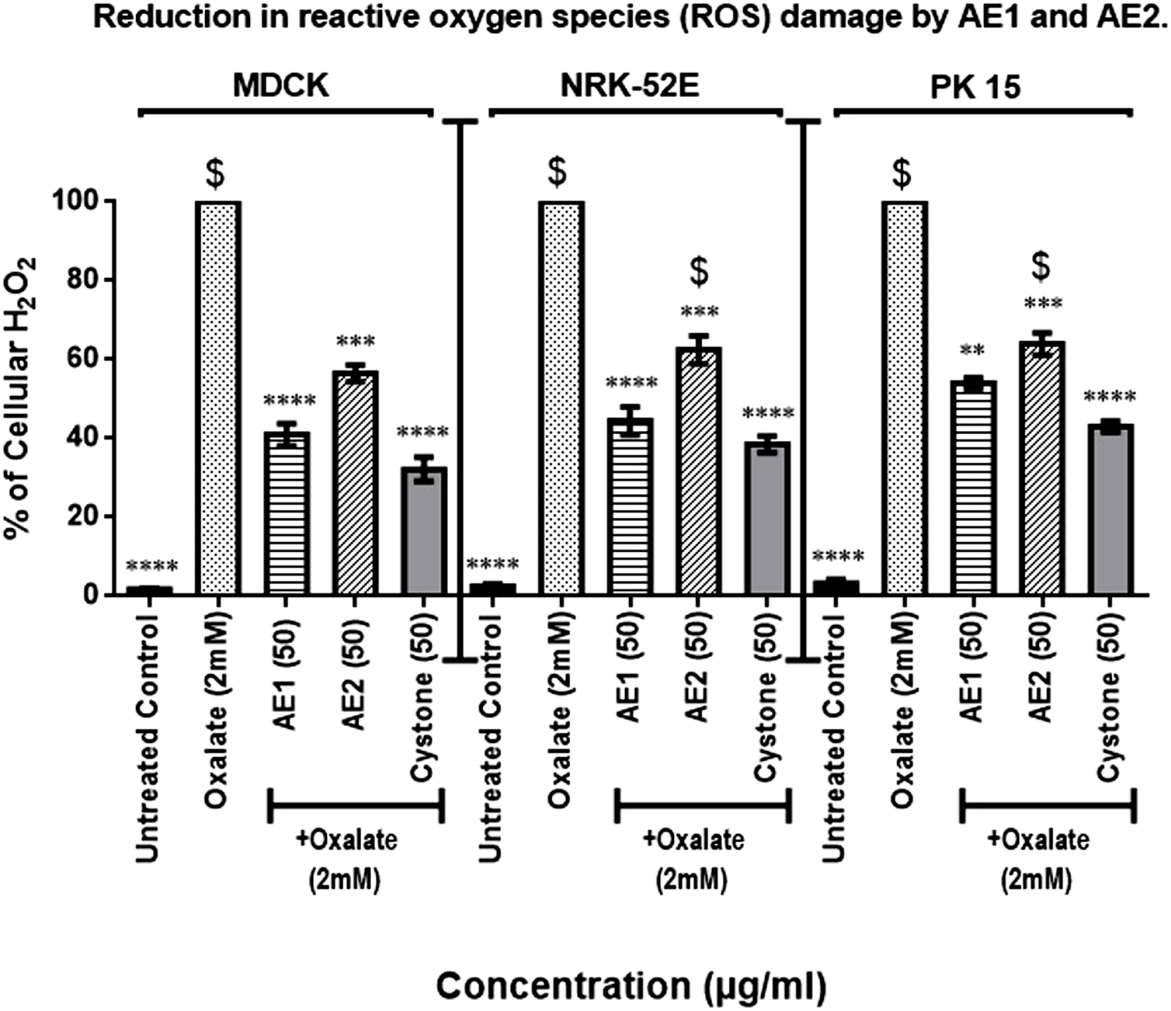
Reduction in percentage of cellular H_2_O_2_ level by statistically optimized aqueous extracts of *T*.*terrestris*. **p<0.01,***p<0.001, ****p<0.0001 showing the significant change w.r.t oxalate treated cells. ^$^p<0.05 indicate the significant difference in percentage of cell viability w.r.t to Cystone treated group *i*.*e* positive control.

To further confirm the ability of the extracts to reduce the oxidative stress owing to oxalate induced injury, the ability to scavenge H_2_O_2_ which is a major component of the ROS pathway and responsible for inducing apoptosis was assessed. Oxalate injury has been reported to induce free radical production and our results were in conformity, wherein, oxalate injured cells exhibited maximum level of H_2_O_2_ production, which was taken as 100%. A dramatic and significant decrease in the cellular H_2_O_2_ levels in oxalate injured cells upon co-treatment with AE1 (50 μg/mL) of 41.5%, 44.5%, 54.2% in MDCK, NRK-52E and PK 15 cells was seen, respectively. Similarly, AE2 (50 μg/mL) treatment also showed significantly reduced levels of cellular H_2_O_2_ production to the tune of 56.5%, 62.5%, 64.3% in MDCK, NRK-52E and PK 15 cells, respectively w.r.t oxalate treated cells (Fig 6). However, it was evident from the results that AE1 was more effective in terms of reduction of H_2_O_2_ production in comparison to AE2. Cystone (50 μg/mL) treatment which served as the positive control, also lead to a significant reduction in cellular H_2_O_2_ in oxalated injured cells, and was comparable to AE1.

### Modulation of crystal morphology and inhibition of oxalate crystal growth by aqueous extracts of *Tribulus terrestris*

**Fig 7:**
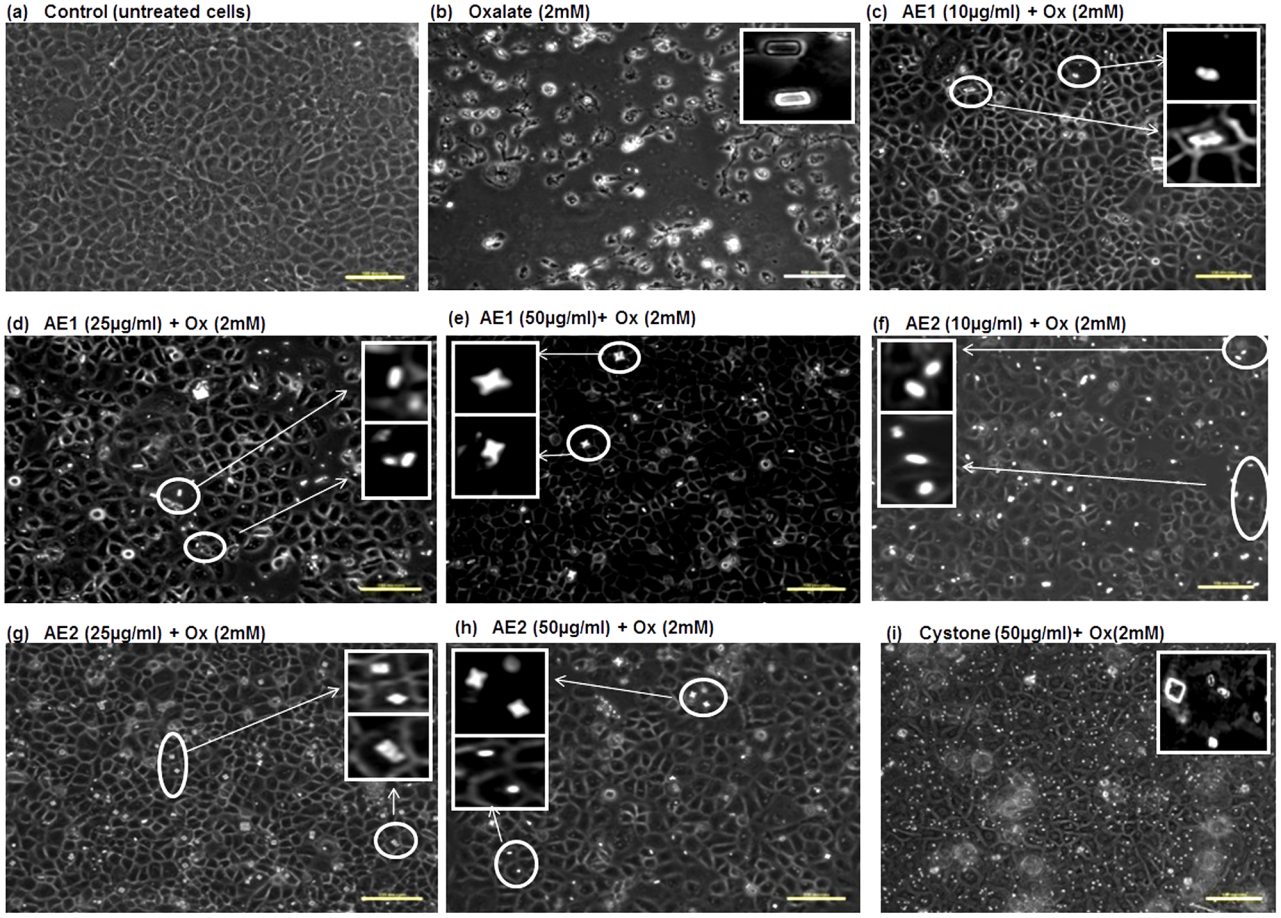
Polarization micrographs: Morphology modulation and inhibition of oxalate crystals growth by statistically optimized aqueous extracts of *T*.*terrestris* on oxalate injured renal epithelial MDCK cells. Polarization microscopic images indicating the morphology modulation efficacy and reduction in crystals adherence potency by AE1 and AE2 in oxalate injured MDCK cells. Cells treated with serum free media considered as untreated control and Cystone (50 μg/mL) were taken as positive control. Images were captured at 20X magnification; scale bar 100 μm. Multiple fields were assessed. Inset images showing the zoomed view of crystals for assessment of shape and morphology of crystals. a: Control b: Injured with 2 mM oxalate c, d and e: treated with AE1 at concentration (10 μg/mL), (25 μg/mL), (50 μg/mL) respectively, and f, g, h treated with AE2 at concentration (10 μg/mL), (25 μg/mL), (50 μg/mL), respectively, i: Cystone treated (50 μg/mL).

**Fig 8:**
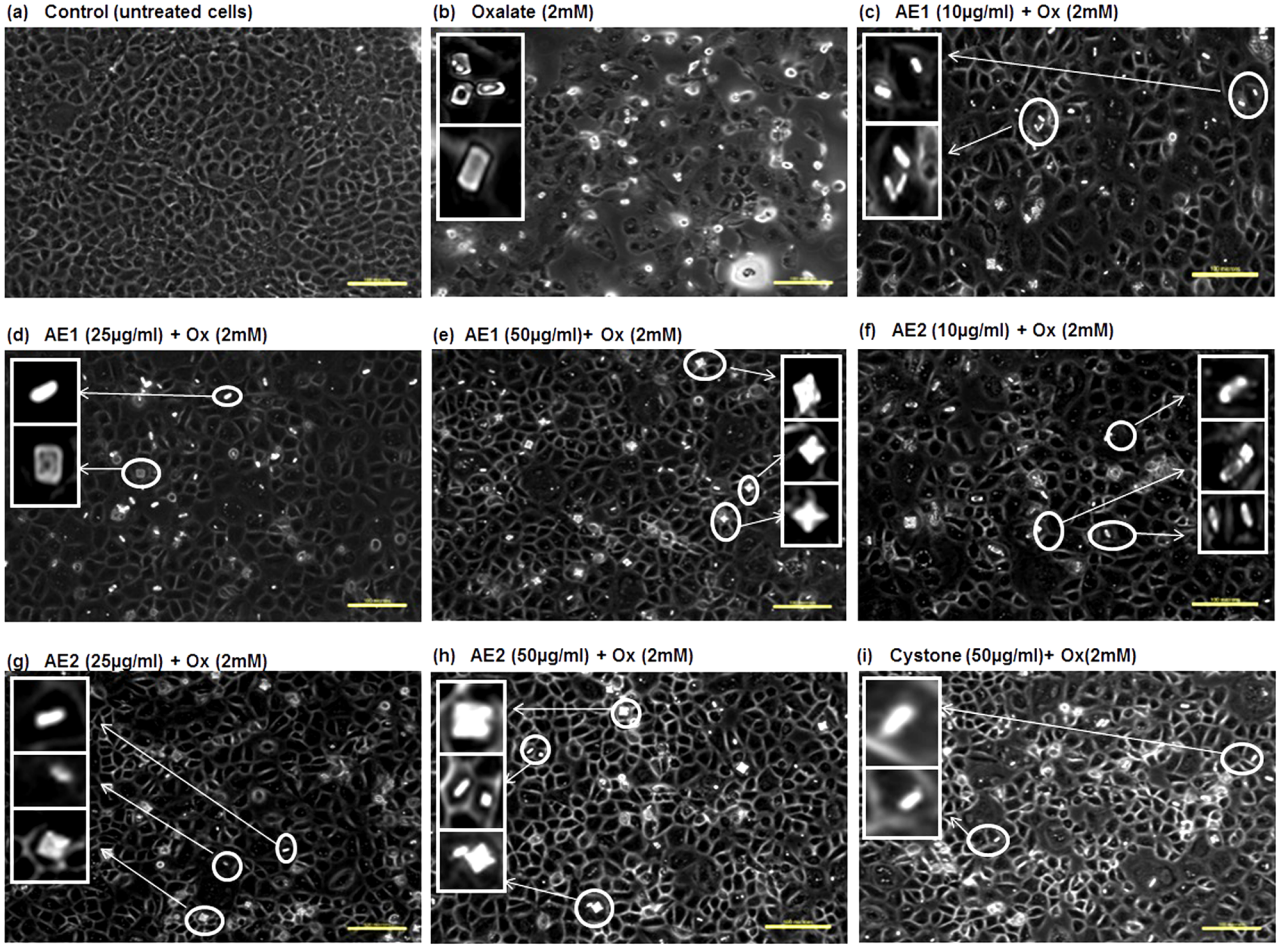
Polarization micrographs: Morphology modulation and inhibition of oxalate crystals growth by statistically optimized aqueous extracts of *T*.*terrestris* on oxalate injured renal epithelial NRK-52 cells. Polarization microscopic images indicating the morphology modulation efficacy and reduction in crystals adherence potency of AE1 and AE2 in oxalate injured NRK-52E cells. Cells treated with serum free media considered as untreated control and Cystone (50 μg/mL) were taken as positive control. Images were captured at 20X magnification; scale bar 100 μm. Multiple fields were assessed. Inset images showing the zoomed view of crystals for assessment of shape and morphology of crystals. a: Control, b: Injured with 2 mM oxalate, c,d and e: treated with AE1 at concentration (10 μg/mL), (25 μg/mL), (50 μg/mL) respectively, and f, g, h treated with AE2 at concentration (10 μg/mL), (25 μg/mL), (50 μg/mL), respectively, i: Cystone treated (50 μg/mL).

**Fig 9:**
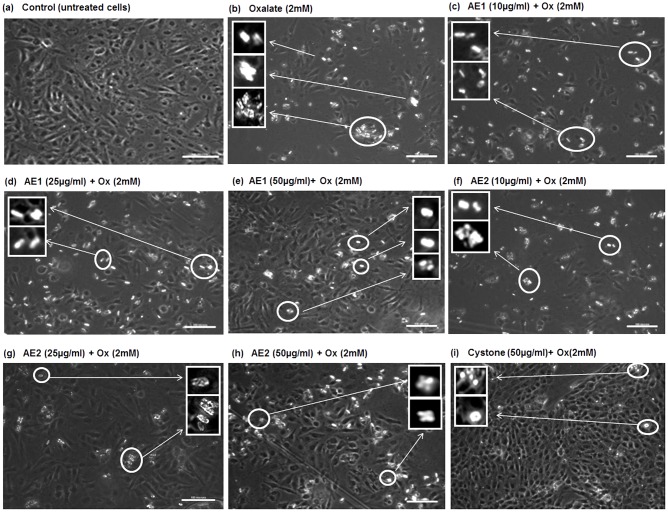
Polarization micrographs: Morphology modulation and inhibition of oxalate crystals growth by statistically optimized aqueous extracts of *T*.*terrestris* on oxalate injured renal epithelial PK 15 cells. Polarization microscopic images indicating the morphology modulation efficacy and reduction in crystals adherence potency of AE1 and AE2 in oxalate injured PK 15 cells. Cells treated with serum free media considered as untreated control and Cystone (50 μg/mL) were taken as positive control. Images were captured at 20X magnification; scale bar 100 μm. Multiple fields were assessed. Inset images showing the zoomed view of crystals for assessment of shape and morphology of crystals. a: Control, b: Injured with 2 mM oxalate, c,d and e: treated with AE1 at concentration (10 μg/mL), (25 μg/mL), (50 μg/mL) respectively, and f,g,h treated with AE2 at concentration (10 μg/mL), (25 μg/mL), (50 μg/mL), respectively, i: Cystone treated (50 μg/mL).

To test whether the extracts obtained had the ability to cause morphological modulation of the COM crystals, thereby attenuating cell- surface interaction, assessment of cell-crystal adhesion by polarization microscopy was done in the cells treated with 10, 25 or 50 μg/mL of AE1 and AE2 for 48 h. Representative polarization micrographs: Fig 7 (MDCK), Fig 8 (NRK-52E) and Fig 9 (PK 15) revealed the cytoprotective efficacy of AE1 and AE2 against oxalate injured cells. Oxalate treatment resulted in cell death due to adhesion of pointed edged COM crystals, as shown in Fig 7b (MDCK), Fig 8b (NRK-52E) and Fig 9b (PK 15). Cell loss due to injury was evident from the diminished cell density and distorted morphology along with increased granularity, as compared to the untreated control shown in Fig 7a (MDCK), Fig 8a (NRK-52E) and Fig 9a (PK 15). Our results showed that both the plant extracts AE1 and AE2 inhibited the oxalate crystal nucleation and aggregation on the surface of renal epithelial cells. As the concentration of AE1 increased from 10 to 50 μg/mL there was a gradual decrease in number along with transition in the morphology of crystals (COM to COD). In addition, intact cellular morphology in MDCK (Fig 7c–7e), NRK-52E (Fig 8c–8e) and PK 15 (Fig 9c–9e) was noted. Similar modifications in the crystal morphology were observed upon treatment with AE2 (10 to 50 μg/mL) in MDCK (Fig 7f–7h), NRK-52E (Fig 8f–8h) and PK 15 (Fig 9f–9h) w.r.t oxalate treated cells. However, observations of polarization micrographs revealed that the AE1(50 μg/mL) was more efficient in modulating the crystals morphology as compared to AE2 as a greater number of COD shaped crystals were seen in MDCK (Fig 7e), NRK-52E (Fig 7e) and PK 15 (Fig 8e) cells as compared to the treatment with AE2 (Fig 7h).

### Microscopic analysis of cell death signals

**Fig 10:**
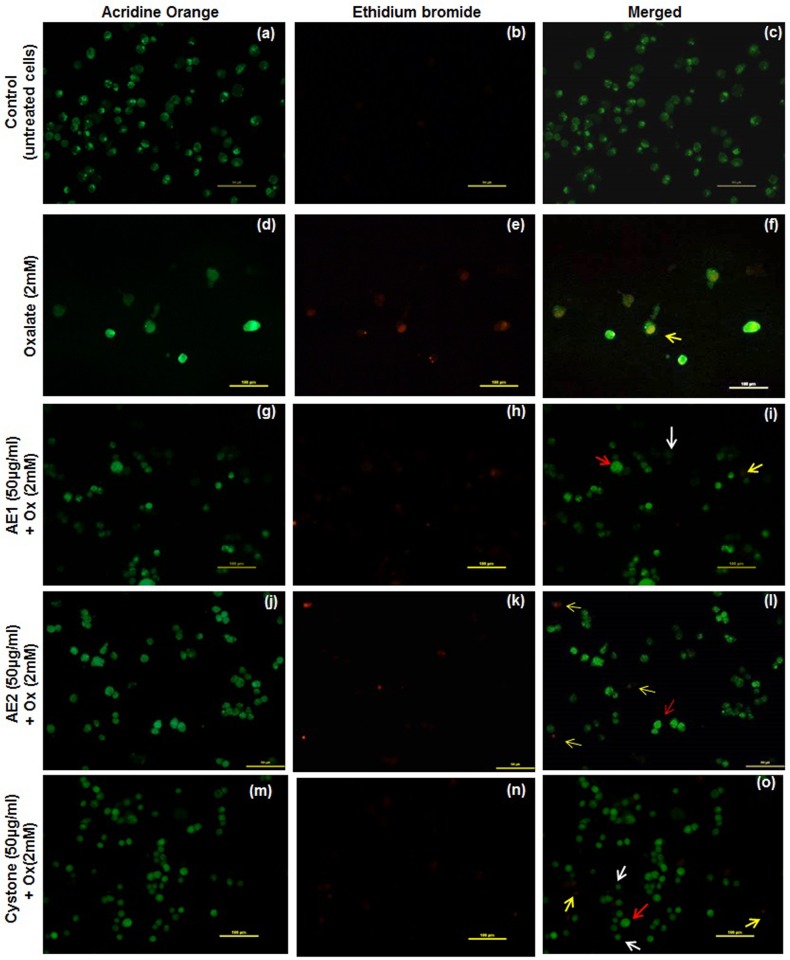
AO/EB dual staining: Reduced programmed cell death signs by statistically optimized aqueous extracts of *T*.*terrestris* in oxalate injured MDCK renal epithelial cells. Detection of apoptosis, necrosis and quantitative changes in dual stained (AO/EB) fluorescence images of MDCK cells. Untreated viable cells were stained a uniform pale green and marked with white arrow, red arrow representing early apoptotic cells (chromatin condensation) were stained bright-green. Late apoptotic cells stained yellow-orange marked by yellow arrow. Cells treated with serum free media were the untreated control. Cystone (50 μg/mL) treatment served as the positive control. Multiple fields were assessed under florescence microscope (Nikon eclipse, Ti) and images were captured at 20 X magnification, scale bar 100 μm. Untreated control- Fig.a: AO stained, Fig.b: EB stained and Fig.c: merged image. Oxalate injury—Fig.d: AO stained, Fig.e: EB stained and Fig.f: merged image. AE1 (50 μg/mL) treatment—Fig.g: AO stained, Fig.h: EB stained, Fig.i: merged image. AE2 (50 μg/mL) treatment—Fig.j: AO stained, Fig.k: EB stained, Fig.l: merged image. Cystone (50 μg/mL)—Fig.m: AO stained, Fig.n: EB stained and Fig.o: merged image.

**Fig 11:**
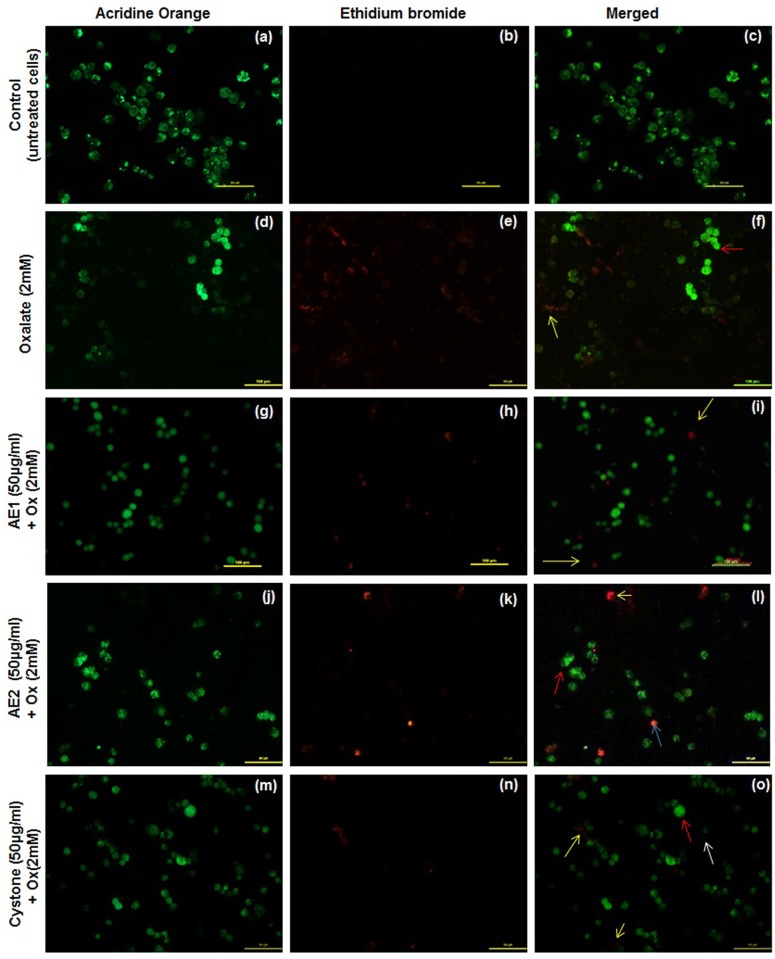
AO/EB dual staining: Reduced programmed cell death signs by statistically optimized aqueous extracts of *T*.*terrestris* in oxalate injured NRK-52E renal epithelial cells. Detection of apoptosis, necrosis and quantitative changes in dual stained (AO/EB) fluorescence images of NRK-52E cells. Viable cells were pale green and marked with white arrow, red arrow represents early apoptotic cells (chromatin condensation) were stained bright-green. Late apoptotic cells stained yellow-orange marked by yellow arrow. Cells showing necrosis stained orange-red and marked with blue arrow. Cells treated with serum free media considered as untreated control. Cystone (50 μg/mL) was taken as positive control. Multiple fields were assessed under florescence microscope (Nikon eclipse, Ti) and images were captured at 20X magnification, scale bar 100 μm. Untreated control- Fig.a: AO stained, Fig.b: EB stained and Fig.c: merged image. Oxalate injury—Fig.d: AO stained, Fig.e: EB stained and Fig.f: merged image. AE1 (50 μg/mL) treatment—Fig.g: AO stained, Fig.h: EB stained, Fig.i: merged image. AE2 (50 μg/mL) treatment—Fig.j: AO stained, Fig.k: EB stained, Fig.l: merged image. Cystone (50 μg/mL)—Fig.m: AO stained, Fig.n: EB stained and Fig.o: merged image.

**Fig 12:**
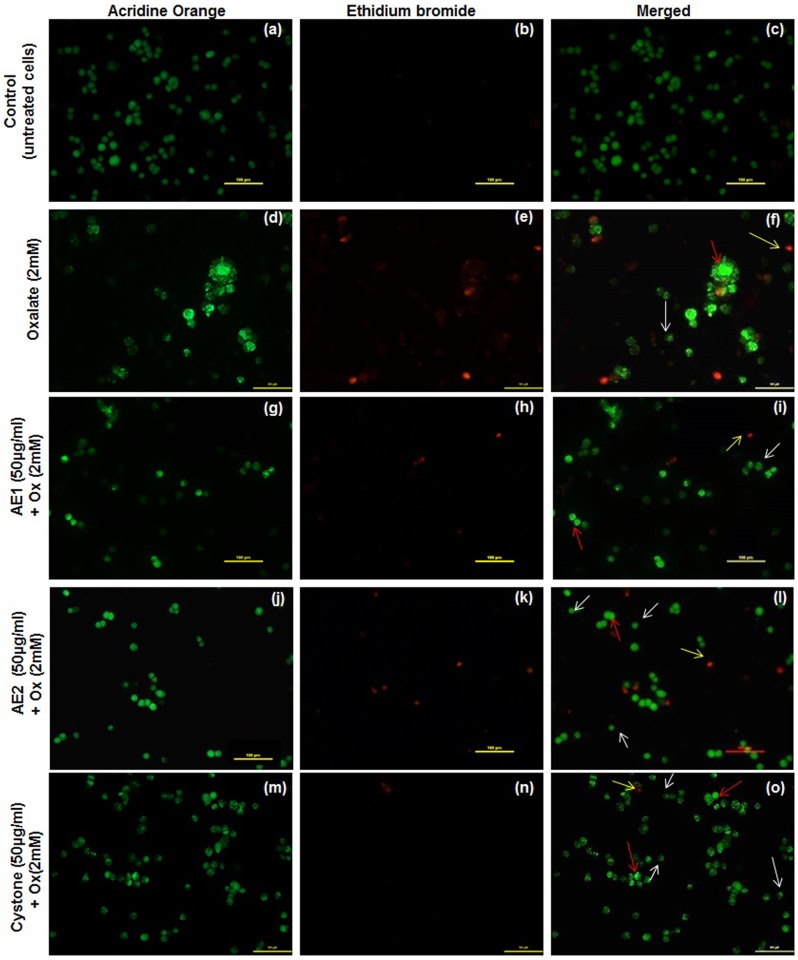
AO/EB dual staining: Reduced programmed cell death signs by statistically optimized aqueous extracts of *T*.*terrestris* in oxalate injured PK 15 renal epithelial cells. Detection of apoptosis, necrosis and quantitative changes in dual stained (AO/EB) fluorescence images of PK 15 cells. Viable cells were pale green and marked with white arrow, red arrow represents early apoptotic cells (chromatin condensation) were stained bright-green. Late apoptotic cells stained yellow-orange marked by yellow arrow. Cells treated with serum free media considered as untreated control. Cystone (50 μg/mL) was taken as positive control. Multiple fields were assessed under florescence microscope (Nikon eclipse, Ti) and images were captured at 20X magnification, scale bar 100 μm. Untreated control- Fig.a: AO stained, Fig.b: EB stained and Fig.c: merged image. Oxalate injury—Fig.d: AO stained, Fig.e: EB stained and Fig.f: merged image. AE1 (50 μg/mL) treatment—Fig.g: AO stained, Fig.h: EB stained, Fig.i: merged image. AE2 (50 μg/mL) treatment—Fig.j: AO stained, Fig.k: EB stained, Fig.l: merged image. Cystone (50 μg/mL)—Fig.m: AO stained, Fig.n: EB stained and Fig.o: merged image.

In order to explore the mode of cell death, which was preliminarily indicated by cell detachment, AO/EB dual staining of the oxalate injured renal epithelial was carried out. Morphological observations from the fluorescent photomicrographs showed that the untreated cells appeared intact and AO dye stained the nuclei uniformly in MDCK (Fig 10a), NRK-52E (Fig 11a) and PK 15 (Fig 12a). However, oxalate injured cells showed typical signs of apoptosis including chromatin condensation, loss of membrane integrity leading to the uptake of the fluorescent dyes and reduction of cell population as represented in Fig 10d, 10e and 10f (MDCK), Fig 11d, 11e and 11f (NRK-52E) and Fig 12d, 12e and 12f (PK 15). Treatment with AE1 and AE2 reduced the hallmarks of apoptosis and necrosis. Furthermore, cytoprotective effects seen in AE1 treated cells were more pronounced which could be attributed to its ability to modulate the crystals as well as scavenge the ROS generated due to injury. AO/EB dual staining observations lend support to the results obtained from cell viability experiments and microscopic (polarization) observations and reiterate the efficacy of AE1.

### Effect of process variables on physicochemical composition of optimized aqueous extracts of *T*.*terrestris*

The medicinal value of any herbal plant is attributed to its phytochemical constituents. Preliminary screening of the RSM optimized extracts (AE1-4) confirmed the presence of major constituents such as saponins, flavonoids, terpenoids, alkaloids as well as proteins. Moreover, our results showed that the extraction condition impacted the amount of extractable material present in the selected aqueous extracts as seen in Table A in S2 Appendix.

On the basis of the antilithiatic potential which was assessed through assays targeting the various steps of the urolithiasis, AE1 showed greater efficacy over the other extracts and was subjected to further phytochemical analysis. Initial characterization of AE1 of *T*. *terrestris* was carried out by Fourier transformer infra-red (FTIR) Spectrophotometry for identification of the functional groups present. Results of FTIR spectra revealed the presence of primary and secondary amines, amides, aldehydes, alcohol, ketone, phenol, esters, alkanes, arenes and alkyl halides (Figure A and Table B in S2 Appendix). The presence of these functional groups thereby validated the presence of secondary metabolites i.e saponins, phenols, flavonoids, tannins, terpenes, proteins, steroids, fatty acids and glycosides which could be attributed towards the therapeutic antilithiatic ability of *T*. *terrestris*.

AE1 was subjected to GC-MS analysis for the identification of compounds by comparing with standard mass spectra in the NIST library. The results revealed the presence of at least 10 different chemotypes representative of the biomolecules present (Figure B in S2 Appendix). Compounds which could contribute to the prevention of kidney stones in terms of either scavengers of free radicals or inhibitors of the inflammatory pathway or as diuretics were detected. The major fatty acid identified was octadecanoic, methyl ester. Saturated fatty acids have been reported to play a role in the regulation of the anti-inflammatory pathway by inhibiting the COX-2 enzyme [21]. Other phytochemicals known to have antioxidant and anti-inflammatory activities which were present were oleic acid, Cholestane, 3,5-dichloro-6-nitro,(3á,5à,6á), Rhodoxanthin, Pregn-4-ene-3,11,20-trione, Lanostane-7,11dione,3,18-bis(acetyloxy),cyclic,. In addition, the presence of compounds having diuretic property such as pyrimidine 2-one and Gamabufotalin could also contribute to the effect of AE1 on the complex process of urolithiasis [22](Table C in S2 Appendix).

## Discussion

For the development of herbal formulation with better clinical efficacy, preliminary screening and characterization of any herbal extract is a necessity. Therapeutic efficacy and potency of herbal formulations are due to synergistic effect of chemical constituents or bioactive compounds present naturally in the herbs [23]. In the light of this, the extraction procedure and parameters must be optimized critically along with the evaluation of *in vitro* cytoprotective capacity to obtain a clinically efficient herbal formulation.

In this study, our first objective was to optimize the extraction procedure statistically through RSM approach. Basically, RSM is a collective package of mathematical and statistical techniques which owing to the ease of use and accuracy, is an important application for the development of new products, process optimization and to improve the quality of existing products [24]. We studied the effect of three variables, temperature, time and solid liquid ratio on the protein content and yield of aqueous extracts of *T*.*terrestris* and then subjected the extracts for evaluation of antilithiatic efficacy. This was done by studying the extent of nucleation and aggregation of CaOx crystals, along with morphology modulation of CaOx crystals using polarization and scanning electron microscopy. Our results from the RSM analysis revealed that the lower temperature and high solid liquid ratio promoted high protein content and higher percent yield. The cumulative effect of temperature and S:L ratio exhibited significant increase in protein content and percent yield, however, combined effect of time and temperature did not significantly affect the yield. Based on the protein content and yield obtained from the RSM optimized aqueous extract of *T*.*terrestris*, four different extracts were selected for further physicochemical screening. As per the guideline provided by WHO [25,26] physicochemical analysis of extracts or formulations is necessary to provide significant information regarding the major constituents present. The presence of phytochemical components in *T*.*terrestris* extracted with various solvents is well documented. Presence of alkaloids, saponins, tannins and glycosides has been reported in methanolic extract [27], ethanolic extract [5] and in aqueous extract of *T*.*terrestris* [28]. A novel antilithiatic *Tribulus terrestris* protein (TTP) was reported by our group [29] which showed cytoprotective efficiency towards oxalate injured renal epithelial cell line NRK-52E. Our results of the phytochemical screening were in concordance with previous studies and revealed the presence of major constituents like tannins, terpenoids, total phenols, *Tribulus terrestris* protein (TTP) and the saponin, tribulosin, in all of the four selected aqueous extracts. Variation in the levels of flavonoids, saponins and alkaloids in AE1, AE2, AE3 and AE4 confirmed that the changes in the extraction parameters impacted the level of extractable components of the extracts. Results of preliminary screening confirmed greater antilithiatic potency of AE1 and AE2 of the bark extracts *of T*.*terrestris* as compared to AE3 and AE4. Presence of antilithiatic protein TTP in AE1, AE2, AE3 and AE4 also confirmed the antilithiatic property of statistically optimized aqueous extracts of *T*.*terrestris* in our study.

Urolithiasis is a complex biological disorder with the critical step being the formation of COM kidney stones due to the conversion of the soluble calcium and oxalate ions to their insoluble crystalline form by the process of crystallization. To be an effective antilithiatic agent it was necessary to validate the ability of the RSM optimized extracts on the process of crystallization. The assays revealed that AE1 and AE2 showed superior inhibitory nucleation and aggregation potential against CaOx as compared to AE3 and AE4.

The next step and probably one of the key ones in this cascade of stone formation is the binding of the COM crystals to the surface of the renal epithelial cells. COM is the most frequently encountered component of urinary calculi and owing to its differential surface charge density as compared to COD, its ability to interact with the renal cell surface is greater. This ability of the COM crystal to adhere to the cells is also intimately dependent on the crystal facets which are presented to the renal cells. These facets can bind to specific sites on the surface thereby attaching to the cells. It has been speculated that under normal conditions these sites are masked and following injury could either be exposed further or increased in number. Based upon the face-selectivity affinity and adhesion kinetics, it has been reported that COM crystals cause greater injury to the renal epithelial cells [30]. Extracts which can therefore cause polymorphism of the COM crystals either by modulating the COM crystal structure or converting it to the less damaging COD would be effective antilithiatic agents. Scientific data has suggested that the extracts of *Bergenia ligulata* [31], *Chenopodium album*[32], *Sesbania grandiflora* [33], *Herniaria hirsute* [34] and *Phyllanthus niruri* [35] have the ability to modulate the COM crystals size as well as number. Modulation of COM crystal morphology by ethanolic extract of *T*.*terrestris* has also been reported [36]. With respect to this, the effect of the 4 extracts obtained from RSM approach were assessed and the results showed a differential capability between the extracts. The morphological changes observed by polarization microscopy confirmed the enhanced capability of AE1 and AE2 of *T*.*terrestris* to alter the COM crystal morphology compared with AE3 and AE4. Moreover, AE1, efficiently modulated the crystal morphology from COM to COD and further also led to a remarkable reduction in the size of COM crystals. Scanning electron micrographs clearly demonstrated the reduction in size, change in shape (COM to COD) and dissolution or blunting of sharp edges of COM crystals which reiterated that the AE1 and AE2 had the ability to protect cells against oxalate injury.

Pathogenic mechanism of kidney stone formation is still unclear but various experimental findings have proved that the CaOx crystal deposition in kidney is the unifying factor which leads to tubulointerstitial damage, inflammation [12], triggers apoptosis, necrosis [37] and causes oxidative stress via ROS damage [38]], which further promotes the aggregation of crystals and perpetuates the damage [30]. Therefore, to be an effective antilithiatic agent the ability to scavenge the free radicals would be desirable and add to its efficacy. Previous studies [39] reported the free radical scavenging activity of methanolic extract of *T*.*terrestris* was due to the presence of phenolic components. To assess whether a similar mechanism was also operative we investigated the free radical scavenging activity of aqueous extracts (AE1, AE2, AE3 and AE4) by DPPH radical scavenging assay and the results confirmed that all the extracts exhibited this property although AE1 had the highest free radical scavenging activity amongst all the four extracts. Furthermore, the GC-MS analysis of AE1 revealed the presence of a number of compounds having the ability to scavenge free radicals, inhibit the inflammatory pathway [22] as well as act as diuretic agents which could be acting in a synergistic manner to diminish urolithiasis damage.

We evaluated the cytoprotective ability of AE1 and AE2 on the oxalate injured renal cells, wherein cells cotreated with AE1 and AE2 exhibited a significant dose-dependent increase in cell viability. As treatment with 50 μg/mL showed the greatest cytoprotective ability in terms of cell viability, for both AE1 and AE2 extracts therefore, we selected this concentration for the subsequent cell culture studies. *In vivo* studies [40], have reported that aqueous extract of *T*.*terrestris* can reduce oxidative stress caused by oxalate injury in Wistar rats. Our results have shown that treatment with AE1 and AE2 extracts, lead to a reduction in ROS damage caused by oxalate injury to renal cells (MDCK, NRK-52E and PK 15). Interaction of renal cells with COM crystals involves attachment of the crystals on the surface of cells followed by internalization [41]. Oxalate injury leads to alteration of cellular physiology and this alteration further impacts attachment of crystals since phosphatidylserine which is normally present on the inner side of the cell membrane, gets exposed on the cell surface, which facilitates the attachment of COM crystals with the renal cells [42]. Results obtained from the *in vitro* crystal adhesion assay in the renal epithelial cells (MDCK, NRK-52E and PK 15) showed protection by AE1 and AE2 extracts against oxalate injured renal cells, as modulation in the shape, size and number of oxalate COM crystals was observed.

To confirm the manner in which cell death was occurring and evaluate the morphological signs, fluorescent microscopic analysis of the treated cells was carried out which showed that AE1 and AE2 both potentially reduced apoptotic and necrotic cell death. Moreover, the observation of similar results in all the 3 mammalian cell lines studied demonstrates that the underlying mechanism could be attributed to the alteration of the crystal morphology from CO M to COD by the extracts. The crystal polymorphism to COD has been reported to be less harmful to renal epithelial cells owing to its lowered affinity to adhere to these cells as compared to the thermodynamically stable COM crystals [43]. Cell culture based *in vitro* experimental data proved the higher antilithiatic property of AE1 as compared to AE2 in concordance with the preliminary screening data. AE1 also exhibited maximum free radical scavenging activity, which is an important factor contributing to its antilithiatic role by reducing the ROS induced damage, programmed cell death, owing to the nucleation and aggregation of oxalate crystals on renal cells.

To date two herbal formulations i.e. Cystone and neeri have been extensively used for the treatment of urolithiasis, however both formulations are composite drugs or we can say combination of number of herbs. Due to unavailability of scientifically statistical optimized data of each herb present in Cystone or neeri it is difficult to hypothesize the percentage of contribution of individual herb or their synergistic effect towards antilithiatic potency in both of these herbal formulations. A key factor in developing any formulation in also authentication of the plant source or the identity, which is easier to determine in a single plant formulation. Moreover we have already shown the presence of a antilithiatic protein TTP in the extracts of *T*.*terrestris* which not only contributes to the antilithiatic activity but also authenticates that the extract has been derived from *T*.*terrestris*. Analysis of the bioactive components present in AE1 by FTIR and GC-MS analysis, throw light on the ability of this extract to act on the various steps of the urolithiatic process by not only modulating the crystal morphology but also contributing to the ability to scavenge ROS and also inhibiting the inflammatory pathway. The use of diuretics for the prevention of recurrent calcium based kidney stones is well documented. The presence of compounds such as pyrimidine 2-one and Gamabufotalin having diuretic properties further lends support to the ability of AE1 to modulate the urolithiasis pathway. Our results show that the bioactive compounds present in a single plant extract of *T*.*terrestris* work in a manner at par with the composite drugs.

## Conclusion

Our data based on several different experimental approaches, establishes that the optimum aqueous extraction condition (temp 23.50°C, time 19.50 h and S: L 1g: 12 mL) derived through RSM lead to the maximum anticalcifying and cytoprotective ability of *T*.*terrestris*. The aqueous extract (AE1) obtained using these extraction conditions showed maximum anticalcifying activity in terms of inhibition of nucleation and aggregation of CaOx crystals. In addition, AE1 exhibited efficacy against urolithiasis, not only in its ability to reduce the size of COM crystals but also in the modulation of the morphology of COM crystals to COD crystals. Apoptosis, necrosis and oxidative stress induced by oxalate injury to renal cells were reduced by AE1 which indicated that the optimized extraction condition had the potential to elicit the maximum antilithiatic potential from the aqueous extract of *T*.*terrestris*. The ability of the optimized *T*.*terrestris* extract to protect renal epithelial cells from injury follows a pathway which appears to be conserved irrespective of the origin of the renal epithelial cells.

As earlier stated the process of kidney stone formation is multifactorial and we have shown that the optimized extract could act on the critical steps in this pathway. Therefore, our study provides the foundation of a single plant extract having antilithiatic potential which is comparable if not superior to known composite drugs. In addition, the data garnered from this study provides useful inputs for the optimization of various parameters which could be further verified through *in vivo* studies. We hope that this study would provide impetus for the development of clinically effective single plant based herbal formulation or single plant based antilithiatic herbal drug in future.

## Supporting information

S1 Appendix **Table A. Combinations of different parameters obtained from central composite design and their experimental and predicted effects on extraction yield (%) and protein content (mg/g).** -ve values were considered as “zero”. **Figure A. Surface quadratic model and coded factor equation for protein.** Showing detailed analysis and significance of statistically optimized model for protein. **Figure B. Surface quadratic model and coded factor equation for yield.** Showing detailed analysis and significance of statistically optimized model for yield. **Figure C. Three dimensional plots (3D) for *T*.*terrestris* showing interaction of different parameters on percent extraction yield.** (a), interaction between temperature (°C) and S: L ratio (g/mL); (b), interaction between incubation time (h) and S:L ratio (g/mL); and (c) interaction between temperature (°C) and incubation time (h). **Figure D. Three dimensional plots (3D) for *T*.*terrestris* showing interaction of different parameters on protein content (mg/g).** (a), interaction between temperature (°C) and S: L ratio (g/mL); (b), interaction between incubation time (h) and S:L ratio (g/mL); and (c) interaction between temperature (°C) and incubation time (h).
(ZIPX)

S2 Appendix **Table A. Physico-chemical analysis of the various extracts obtained through RSM.** # Results expressed in mg/g. **Table B. Fourier transform infra-red (FTIR) analysis.** cm^-1^ vibrations. **Table C. Major compounds in AE1 by GC MS analysis. Figure A. Fourier transform infrared spectroscopy (FTIR) spectrum of statistically optimized aqueous extract AE1 of *Tribulus terrestris* in frequency range 400–4000 cm**^**-1**^**. Figure B. Metabolic profiling by GC-MS analysis of statistically optimized aqueous extract AE1 of *Tribulus terrestris*.**
(ZIPX)

## Abbreviations

AE: Aqueous extract
CaOx: Calcium oxalate
COD: Calcium oxalate dihydrate
COM: Calcium oxalate monohydrate
dAE: Deproteinated aqueous extract
DPPH: 1,1-diphenyl-2-picrylhydrazyl
MDCK: Madin-Darby Canine Kidney
NRK-52E: Normal rat epithelial Kidney
PCA: Perchloric acid
PK 15: Porcine Kidney
ROS: Reactive oxygen species
RSM: Response surface methodology
S:L: Solid to liquid ratio
TTP: *Tribulus terrestris* protein
